# Absence of *A3Z3*-Related Hypermutations in the *env* and *vif* Proviral Genes in FIV Naturally Infected Cats

**DOI:** 10.3390/v10060296

**Published:** 2018-05-31

**Authors:** Lucía Cano-Ortiz, Dennis Maletich Junqueira, Juliana Comerlato, André Zani, Cristina Santos Costa, Paulo Michel Roehe, Ana Cláudia Franco

**Affiliations:** 1 Virology Laboratory, Institute of Basic Health Sciences, Federal University of Rio Grande do Sul, Rua Sarmento Leite 500, Porto Alegre, RS CEP 90150-070, Brazil; jucomerlato@gmail.com (J.C.); zani.andre@yahoo.com.br (A.Z.); cscostaa@gmail.com (C.S.C.); proehe@gmail.com (P.M.R.); anafranco.ufrgs@gmail.com (A.C.F.); 2 Health Science Department, UniRitter Laureate International Universities, Rua Orfanotrófio, 555, Alto Teresópolis, Porto Alegre, RS CEP 90840-440C, Brazil; dennismaletich@hotmail.com

**Keywords:** APOBEC3, FIV, hypermutations, cats

## Abstract

Apolipoprotein B mRNA-editing enzyme catalytic polypeptide-like 3 (APOBEC3; A3) proteins comprise an important family of restriction factors that produce hypermutations on proviral DNA and are able to limit virus replication. Vif, an accessory protein present in almost all lentiviruses, counteracts the antiviral A3 activity. Seven haplotypes of *APOBEC3Z3* (*A3Z3*) were described in domestic cats (hap I–VII), and in-vitro studies have demonstrated that these proteins reduce infectivity of vif-defective feline immunodeficiency virus (FIV). Moreover, hap V is resistant to vif-mediated degradation. However, studies on the effect of *A3Z3* in FIV-infected cats have not been developed. Here, the correlation between APOBEC *A3Z3* haplotypes in domestic cats and the frequency of hypermutations in the FIV *vif* and *env* genes were assessed in a retrospective cohort study with 30 blood samples collected between 2012 and 2016 from naturally FIV-infected cats in Brazil. The *vif* and *env* sequences were analyzed and displayed low or undetectable levels of hypermutations, and could not be associated with any specific *A3Z3* haplotype.

## 1. Introduction

Feline immunodeficiency virus (FIV) is a lentivirus of the *Retroviridae* family which is able to infect several species of *Felidae*. It has been associated with feline acquired immunodeficiency syndrome (FAIDS) in domestic cats (*Felis catus*) [1]. Besides its biological and genomic similarity with HIV, which makes it a valuable natural model for the study of AIDS [2], FIV has significant veterinary importance due to its high prevalence in domestic cats worldwide [3]. As characteristic to retroviruses, one of the steps of the FIV replication cycle is the reverse transcription of the genome; the reverse transcriptase enzyme uses the viral RNA as a template for the provirus synthesis, which will be further integrated in the genome of the host cell. The proviral DNA is flanked by long terminal repeats (LTRs) and is constituted by structural genes (*gag*, *pol* and *env*) and accessory, auxiliary and regulatory genes (*vif*, *orfA* and *rev*, respectively) [3]. Among the proteins encoded by these genes, the viral infectivity factor (vif) is a 23-29 kDa protein essential to the formation of infectious viral particles in nonpermissive cells [4,5,6].

Mammalian cells express restriction factors that play an important antiretroviral role in innate immunity. One of them is apolipoprotein B mRNA-editing enzyme, catalytic polypeptide-like (APOBEC3, A3), which belongs to the family of the DNA cytidine deaminases [7]. Cats encode four APOBEC3 proteins (A3Z2a, A3Z2b, A3Z2c and A3Z3). In addition, a fifth protein, named A3Z2Z3, is expressed by read-through alternative splicing [8]. The A3 proteins expressed in infected cells are encapsulated in the virions and act in the cytoplasm of the target cell, after RNA liberation [9,10]. During the reverse transcription of the viral genome, A3 catalyzes a deamination reaction by converting cytidines into uridines, thus inducting G-to-A hypermutations in the newly synthesized viral DNA [11,12,13]. As a viral countermeasure, A3 activity is drastically diminished as it is tagged for proteosomal degradation by vif, encoded by the virus genome [14,15].

The interaction among vif, A3 and a ubiquitin ligase complex leads the polyubiquitination and degradation of A3 proteins [16,17]. Vif contains different N-terminal motifs that interact with A3Z2 and A3Z3. Moreover, it is able to interact through the residues 50–80, outside the interaction sites [18]. Importantly, alterations in the coding sequence of A3 may modify the stability, subcellular localization and, consequently, its interaction with vif. Seven haplotypes of *A3Z3* encoded by domestic cats have been reported [19]. Among them, only haplotype V has demonstrated resistance in vitro to FIV vif-mediated degradation, determined by the lateral chain of the amino acid on position 65 [20]. The objective of this retrospective cohort study was to correlate the effect of different *A3Z3* haplotypes on the frequency of hypermutations in the *env* and *vif* gene sequences of cats that tested FIV positive by the SNAP FIV/FeLV Combo Test (Idexx) or PCR.

## 2. Materials and Methods

### 2.1. Samples and DNA Extraction

Thirty samples of peripheral blood from FIV naturally infected cats from Porto Alegre, RS, Brazil collected between 2012 and 2016 were used for the analyses. The DNA extraction was performed using buffer-saturated phenol and the DNA was stored at −20 °C. Animals were nonpedigree cats, characterized by being a genetically homogeneous population. All the study protocols were approved by the Ethics Committee on Animal Use (CEUA) of the Federal University of Rio Grande do Sul (UFRGS). Project number 29749, permission date 4 October 2016.

### 2.2. A3Z3, env and vif Amplifications and Sequencing

Exon 3 of the *A3Z3* gene (coding for APOBEC3) was submitted to a PCR with primers A3H2F and A3H3R, as described previously [19]. FIV provirus was amplified by a nested PCR. The first round of amplification was performed with primers VIF_FIV_PF and ENV_PR [18] generating a 3.1-kb-long fragment using the Phusion High-Fidelity DNA Polymerase (New England Biolabs, Ipswich, MA, USA). In order to amplify part of the *env* gene, a second round of amplification was made with primers ENV2-3_PF and ENV2-3_PR [21], giving rise to an expected final product of 831 pb. In order to amplify the *vif* gene, the 3.1 kb was submitted to another second round of amplification with primers VIF_FIV_PF and VIF_FIV_PR [18], generating a 756-pb-long fragment that encompassed the entire *vif* gene (sequences of primers are available in the Appendix data, Table A1). In order to sequence the entire *vif* gene, this product was cloned into pCR2.1 vector using TOPO TA Cloning kit (Thermo Fisher Scientific, Waltham, MA, USA). Final PCR products of *A3Z3* (590 pb), *env* (831 pb) and cloned fragment of *vif* were sequenced using BigDye Terminator v3.1 Cycle Sequencing (Applied Biosystems, Waltham, MA, USA). Three *vif* clones per sample were sequenced. The generated chromatograms were then assembled using the Geneious^®^ software (version 9.0.5, Auckland, AUK, NZ, http://www.geneious.com) [22].

### 2.3. Genotype and Haplotype Analyses

Genotypes and allele frequencies were determined manually by gene counting in *A3Z3* gene. The Hardy–Weinberg equilibrium was calculated. The haplotype was determined and analyzed with the program MLOCUS [23]. The linkage equilibrium was also calculated.

### 2.4. Hypermutations Analyses

**Figure A1:**
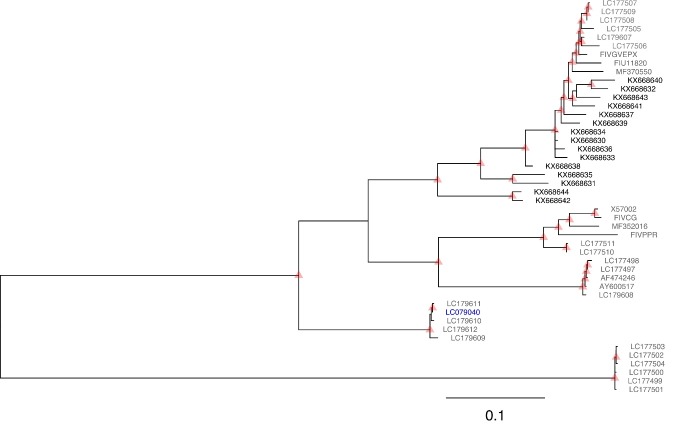
Mid-point rooted maximum-likelihood phylogenetic tree of 46 samples from feline immunodeficiency virus (FIV) *vif*. Bold tips mark the sequences described in this study. The tip marked in blue was used as reference sequence in the hypermutation analyses. Red triangles at the nodes mark Approximate Likelihood-Ratio Test (aLRT) support values >70. Branch lengths are drawn to scale with the bar at the bottom indicating nucleotide substitutions per site.

The *env* and *vif* sequences were submitted to analyses with the program Hypermut 2.0. Such program identifies G→A hypermutations using the default setting, where hypermutations are detected in a GRD motif (where R is code for G or A, and D for G, A or T), and the context requirements are enforced on query sequences. To select the reference sequence for the analyses of the *env* gene fragment, a phylogenetic reconstruction was performed using parameters and reference described in our previous work [21]. Briefly, we submitted our dataset (including 50 sequences from public databases and 27 sequences described in this study) to a maximum-likelihood phylogenetic reconstruction in PhyML. Our 27 sequences grouped in a monophyletic clade inside subtype B group. As the reference sequence should represent ancestral characters for hypermutation analyses in Hypermut, a reference sample was searched in branches at the basal positions of clade generated by the 27 sequences here reported (D37812). For *vif* analyses, a phylogenetic tree was reconstructed using 46 sequences (including our 15 *vif* sequences generated in this study) by the same method described above. Sequence LC079040, which belongs to a sister group of the cluster of sequences described in this study, was used as reference (Figure A1). In addition, several other attempts to analyze hypermutions in *env* and *vif* sequences using different reference sequences were performed. Hypermut is available from http://www.hiv.lanl.gov/content/sequence/HYPERMUT/hypermut.html [24].

## 3. Results

### 3.1. PCR Amplification and Sequencing

The *A3Z3* exon 3 was successfully amplified and sequenced from all of the 30 samples examined in the present study; *env* partial gene sequences were obtained from 27 samples; complete *vif* sequences were obtained from 15. The Genbank accession numbers for partial *env* sequences are MF062041-MF062051, MF062053-MF062055 and MF062057-MF06206, and for the complete *vif* genes the numbers are KX668630–KX668644.

### 3.2. Identification of A3Z3 Haplotypes

In the present study, five out of the seven previously described haplotypes were detected among the 30 samples here examined [23]. The frequencies of each of the genotypes are shown in Table 1. The occurrence of such haplotypes was evidenced in both chromosomes in sampled animals, totalizing 60 haplotypes. Haplotype GGGG was the most frequently detected (30 times, see Table 2).

### 3.3. Detection of Hypermutations

Although variation in provirus sequences was found between animals, both groups of sequences (*env* and *vif*) showed low or undetectable levels of hypermutations (*p* > 0.05). This was also true among the sequences from the cat with *A3Z3* haplotype V, preventing the correlation between the A3 haplotypes and hypermutations. The same results were obtained when other sequences were used as the reference.

## 4. Discussion

According to a previous study on naturally occurring *A3Z3* haplotypes, the polymorphisms in the exon 3 of feline *A3Z3* gene are as follows: A65S (A65I), R68Q, A94T and V96I [20]. The frequencies of *A3Z3* haplotypes found here were not different from the ones described by Castro et al. (2014): *p* = 0.6257 (Table 2). The calculated linkage disequilibrium between polymorphisms indicated that all loci segregate together (values of D′ < 0.18). However, unlike the study of Castro et al., we did not detect haplotypes VI and VII in our cat population, most likely due to the low frequency of these variants [19]. Possibly such haplotypes would be represented among a larger number of samples. In the present study, only one sample belonged to *A3Z3* haplotype V, whose frequency in the cat population has previously been described as low (3.7%) [19]. Haplotype V was previously correlated with resistance to FIV infection [19], and was resistant to vif-mediated degradation in vitro [20]. 

In order to find a correlation between the *A3Z3* haplotypes, the proviral sequences and a resistance to vif-mediated degradation in *env* and *vif* genes, the sequences of *env* and *vif* were analyzed with Hypermut 2.0 sofware, searching for G→A hypermutations. All *vif* sequences obtained in each sampled animal were identical, confirming their nucleotide sequence and thus, no correlation was found in this study between *A3Z3* haplotypes and hypermutations in the target regions of the viral genome. However, these results might have been influenced by the relatively small number of samples/sequences analyzed. In humans, the effect of different SNPs in the function of A3 has been more broadly studied, and A3 variants were associated with hypermutations in *env*, *gag* and *pol* and with the progression of the disease in HIV-positive patients. For instance, a previous study with antiretroviral-therapy-naive women (*N* = 28) found 16 individuals with hypermutated *gag* sequences. However, only 20 out of 373 *gag* sequences of these patients were hypermutated [25]. In another report, APOBEC3G- and APOBEC3F-associated hypermutations were detected in 12 and three out of 127 patients, respectively, and such hypermutations in HIV were strongly associated with a defective *vif* [26].

Although some of the previously mentioned studies have relied on DNA amplification, cloning and single-clone sequencing, as performed here, such methodology may lead to inconclusive results because, in principle, such mutated viruses are in disadvantage in comparison to the original viruses, and hypermutated sequences are usually found in small numbers [27].

Here, the sample used to search for hypermutations in FIV-positive cats was peripheral blood. Hypermutated proviral HIV genomes have been previously detected from PBMC in 39%, 40% and 8% of patients with undetectable viral load in the absence of antiretroviral therapy (elite controllers), treated controls and untreated controls, respectively (*N* = 46) [28]. However, using alternative tissues may also help with finding such mutations, as in HIV-positive patients they have more easily been found in sanctuaries (cerebral spinal fluid and rectal tissue) of HIV than in PBMC [29].

The study of other *A3Z3* regions or other feline A3 genes may help to establish a correlation with resistance or susceptibility to the virus in vivo and possibly with the occurrence of hypermutations in different FIV genes. Further, it is possible that the identification of the nucleotide sequences of the whole *env* gene, as performed in other studies with HIV or other genes, such as *pol* and *gag*, would clarify the relation between *A3Z3* and the occurrence of hypermutations in the FIV genome [30,31]. Remarkably, hypermutations in two HIV-positive patients in *env* and *vpu* genes were not associated with mutations in *vif* [29]. In line with this, in the present study some polymorphisms were observed in the *vif* sequences, but none of them in regions important for interactions with *A3Z3* and *A3Z2* [18]. The sequences of *vif* showed that all of those are functional, and do not present deleterious mutations that could impair their function. This is evidenced by the lack of hypermutations detected in the *env* and *vif* genes. 

This study cannot provide any solid conclusion, as the number of samples analyzed is small (and the genomic regions of the virus analyzed are also limited). Hence, the study failed to find any association between A3 variants and hypermutations in the FIV genome. On the other hand, we cannot rule out that there is an association, as the power of the study may be too small to reveal it. Further studies using a larger number of samples from FIV-infected cats will be necessary to better understand the association between A3 variants and clinical status, virus load and other parameters, as well as the role of other restriction factors in the progression of FIV infections in domestic cats.

## Figures and Tables

**Table 1:** Polymorphisms, genotypes and frequencies in the *A3Z3* from feline immunodeficiency virus (FIV) positive cats in Porto Alegre city.

Polymorphism	Codons	Genotype	Animals *N* = 30 (%)
A65S	TCA/TCA TCA/GCA GCA/GCA GCA/ATA	TT TG GG GA	5 (16.7%) 18 (60%) 5 (16.7%) 2 (6.7%)
R68Q	CAG/CGG CGG/CGG	AG GG	10 (33.3%) 20 (66.7%)
A94T	ACG/GCG GCG/GCG	AG GG	10 (33.3%) 20 (66.7%)
V96I	ATG/GTC GTC/GTC	AG GG	4 (13.3%) 26 (86.7%)

**Table 2:** Haplotypes and frequencies of the gene *A3Z3* found in the studied population of FIV-positive cats in Porto Alegre city, and comparison of frequencies with the previous study in the same conditions.

Number [20]	Haplotype	Frequency (N)	Frequency in the Same Population (N) [19]
I	GGGG	0.5 (30)	0.55045871 (120)
II	TGGG	0.23 (14)	0.26605505 (58)
III	TAAG	0.17 (10)	0.09174312 (20)
IV	TGGA	0.07 (4)	0.0412844 (9)
V	AGGG	0.3 (2)	0.03669725 (8)
VI	GGGA	0 (0)	0.00917431 (2)
VII	TAGG	0 (0)	0.00458716 (1)
	Total	1 (60)	1 (218)

## Appendix A

**Table A1:** Primers used in this study to amplify proviral genes (*env* and *vif*) from exon 3 of *A3Z3*-positive FIV cats.

Region	Primer Name	Sequence (5′–3′)	Size
Exon 3 *A3Z3*	A3H2F	TCATCCCCAATGGCACCCACAGC	590 pb
	A3H3R	TCAAACTCTGAGACGGAGGAGGAG	
External primers	VIF_FIV_PF	CTTCCTGAAGGGGATGAGTG	3.1 kb
	ENV_PR	CCTARTTCTTGCATAGCRAAAGC	
Internal primers vif	VIF_FIV_PF	CTTCCTGAAGGGGATGAGTG	756 pb
	VIF_FIV_PR	ATCTCTTCCATTCATAGYTCTCC	
Internal primers env	ENV2-3_PF	GAATGAGACTATAACAGGAC	831 pb
	ENV2-3_PR	CAAGACCAATTTCCAGCAAT	
